# Treatment of wheat straw using tannase and white-rot fungus to improve feed utilization by ruminants

**DOI:** 10.1186/2049-1891-5-13

**Published:** 2014-02-20

**Authors:** Shailendra Raghuwanshi, Swati Misra, Rajendra Kumar Saxena

**Affiliations:** 1 Department of Microbiology, University of Delhi South Campus, Benito Juarez Road, New Delhi 110 021, India

**Keywords:** *Ganoderma* sp, High energy cattle feed, Tannase, Tannin free myco-straw

## Abstract

**Background:**

Current research to enrich cattle feed has primarily focused on treatment using white rot fungi, while there are scarce reports using the enzyme tannase, which is discussed only in reviews or in the form of a hypothesis. In this context, the aim of the present study was to evaluate the effect of tannase on wheat straw (WS) and also the effect of lyophilized tannase at concentrations of 0.1%, 0.2%, and 0.3% (w/w) on WS followed by fermentation with *Ganoderma* sp. for 10 d and compared in relation to biochemical parameters, crude protein (CP) content, and nutritional value by calculating the C/N ratio in order to improve the nutritional value of cattle feed.

**Results:**

*Penicillium charlesii,* a tannase-producing microorganism, produced 61.4 IU/mL of tannase in 54 h when 2% (w/v) tannic acid (TA) was initially used as a substrate in medium containing (% w/v) sucrose (1.0), NaNO_3_ (1.0), and MgSO_4_ (0.08 pH, 5.0) in a 300-L fermentor (working volume 220 L), and concomitantly fed with 1.0% (w/v) TA after 24 h. The yield of partially purified and lyophilized tannase was 5.8 IU/mg. The tannin-free myco-straw at 0.1% (w/w) tannase showed 37.8% (w/w) lignin degradation with only a 20.4% (w/w) decrease in cellulose content and the *in vitro* feed digestibility was 32.2%. An increase in CP content (up to 1.28-fold) along with a lower C/N ratio of 25.0%, as compared to myco-straw, was obtained.

**Conclusions:**

The use of tannin-free myco-straw has potential to improve the nutritional content of cattle feed. This biological treatment process was safe, eco-friendly, easy to perform, and was less expensive as compared to other treatment methods.

## Background

The drastic increase in human population has caused intensified pressure on agricultural land use, which has led to the conversion of farmland into commercial landscapes. Therefore, countries with agriculture-based economies have endured economic constraints that have negatively impacted the health of cattle due to nutritional deficiencies of feed. Therefore, it is of the utmost importance to improve the quality of cattle feed so that it can be easily be digested by ruminants; therefore, producing higher milk yields to benefit the ever-increasing demand for milk and milk products. Therefore, to overcome problems associated with the decrease in available farmland and the increased demand for milk and milk products, efforts are being made to reduce the wide gap between the requirement and availability of feed and fodder, including technological interventions to increase yield, expand areas to raise fodder crops, conserve land for growing feed and fodder, improve the nutritional value of poor quality roughages, and develop unconventional feeds at competitive prices [1]. The main focus of cattle feed research has been on the enhancement of the nutritional value of dry roughages using physical, chemical, and biological treatments [2].

Globally, India is the second largest producer of wheat as a cereal crop, which, incidentally, produces a large amount of crop residue. Apart from feed and fodder production, a very limited amount of wheat straw (WS) is used for various other purposes such as the manufacture of ethanol, paper, and fertilizer, while the majority is either removed from the field, burned on site, piled, spread over fields, or used as mulch [3]. However, the burning of straw causes atmospheric pollution, although it is a cost-effective disposal method [4]. Although the use of WS for ruminant feeding is constrained by its low digestibility of the energy-rich cell walls because of the high silica content, the tannins, lignin, and the relatively low protein and energy content, when fed to cattle as the only feed source it could significantly influence livestock production [5,6].

Several physical and chemical treatments have been proposed to improve the degradability of WS and subsequent feed intake by cattle. Still, the practical use of these treatments is restricted by safety concerns, cost, and potentially negative environmental impacts [7]. In this respect, an alternative approach is the use of ligninolytic fungi or their enzymes to shorten the incubation period and reduce the use of potentially toxic chemicals [3]. Enzymatic and biological delignification has an edge over other treatment methods to improve the nutritional value of WS, as it includes mild reaction conditions, avoids the use of toxic and corrosive chemicals, produces higher product yield, and has fewer side reactions, lower energy demand, and less reactor resistance [8-10]. For biological delignifiaction, white rot fungi (WRF), a type wood-decaying basidiomycetes, act as lignocellulolytic microorganisms, which contains a group of enzymes of varied functions, including the hydrolytic enzymes cellulase and xylanases that degrade polysaccharides and oxidative ligninolytic enzymes such as lignin peroxidase, manganese peroxidase (MnP), and laccase that degrade lignin. Several WRF species are effective lignin degraders that can be exploited to improve the nutritional value of fodder for ruminant nutrition without compromising the cellulose and hemicellulose constituents [11-13].

Tannin acyl hydrolase, commonly referred to as tannase (E.C. 3.1.1.20), is produced by various micro-organisms, and has been reported to improve the nutritional value of feed by the degradation of tannins. It is also able to hydrolyze ester bonds (i.e., the galloyl ester of an alcohol moiety), as well as depside bonds (the galloyl ester of gallic acid, GA) of substrates (or complex polyphenols) such as tannic acid (TA), epicatechin gallate, epigallocatechin gallate, and chlorogenic acid [14]. The breakdown of complex polyphenols could lead to production of GA, epicatechin, epicatechin gallate, and glucose [15]. To date, studies on feed enrichment have mainly focused on treatment using WRF, whereas reports using tannases remain scarce and are presented only in reviews or in the form of hypotheses.

In the present study, the effect of tannase-treatment on WS subjected to fungal pellet inoculum of *Ganoderma* spp. was examined and compared in relation to biochemical parameters and crude protein (CP) production to improve the nutritional value of cattle feed, as evaluated by the carbon-to-nitrogen (C/N) ratio.

## Materials and methods

### Chemicals and reagents

All chemicals used in this study were of commercial/analytical reagent grade and purchased from Sisco Research Laboratories Pvt., Ltd. (Mumbai, India), Central Drug House Pvt., Ltd. (Mumbai, India) or SS Fermozyme Pvt. Ltd. (New Delhi, India).

### Estimation of tannase content

The spectrophotometric method described by Deschamps et al. [16] was used to estimate tannase activity. One IU of tannase was defined as the amount of enzyme required to release 1.0 μmol of GA/mL of the substrate in 1 min under the specific assay conditions.

### Estimation of residual TA content

The remaining TA content in the fermented broth was estimated using the methods of Hagerman and Butler [17] and expressed as a percentage of the initial concentration. The effect of partially purified and lyophilized tannase on the nutritional value of cattle feed (WS) was evaluated.

### Application of tannase to improve the nutritional value of cattle feed (WS)

#### ***Preparation of tannin-free WS***

Pre-dried WS was treated with different concentration of tannase (0.1%–0.3% (w/w) with an activity of 5.8 IU/mg) to remove tannins. Briefly, lyophilized tannase was dissolved in citrate phosphate buffer and sprayed on WS to obtain a moisture ratio of 1:2 (WS: moisture) and then mixed, incubated at 40°C under static conditions for 6.0 h, and analyzed for remaining tannin content.

The untreated and enzyme-treated WS samples were powdered using a laboratory mill (Remi Motors, Delhi, India) and sieved (30 mesh, 0.5 mm) for analytical purposes. After enzyme treatment, the WS was considered tannin-free.

### Experimental organisms and inoculum preparation

*Ganoderma* spp. obtained from a laboratory stock culture collection (isolated from the bark of *Eucalyptus lanceolate* from the forested area near Delhi, India), were maintained on malt extract agar (MEA), containing (g/L) malt extract, 20.0; KH_2_PO_4_, 0.5; MgSO_4_•7H_2_O, 0.5; Ca(NO_3_)_2_•4H_2_O, 0.5; and agar, 20.0 (pH 5.5), at 30°C. The obtained culture was stored at 4°C and subcultured every 2 wk. A 250-mL Erlenmeyer flask containing 50 mL of sterile malt extract broth (MEB) was inoculated with two mycelial discs (8 mm in diameter) from the periphery of 7 d-old fungal cultures grown on MEA. The inoculum was incubated at 30°C for 5 d under static conditions. The obtained mycelial mat was further homogenized and then inoculated (4% v/v) in 2-L Erlenmeyer flasks containing 500 mL MEB and incubated at 30°C for 5 d in a rotary incubator at 200 rpm. The fungal mass, obtained in the form of pellets, was separated from the culture broth through filtration using pre-weighed Whatman grade no. 1 filter paper. The formed pellets were used as inocula for further experiments. After being thoroughly washed with deionized water, dried at 55°C until a constant mass was achieved, and weighed.

### Solid state fermentation

Enamel trays (40 cm × 32 cm × 7.5 cm) were loaded with 500 g of tannin-free or untreated WS (as a control) and moistened with mineral salt solution and sterilized. Each sterilized tray was inoculated with a dry fungal mass of 3.75–4.00 g (0.75% w/w). Sterile mineral salt solution was added to the trays to obtain a final substrate-to-moisture ratio of 1:3 and incubated at 30°C under 60% relative humidity for 10 d. Following incubation, the trays were harvested to determine production of various enzymes and the proximate and chemical composition of the WS. All experiments were performed in three times.

### Enzyme assays

The myco-straw (WS, both enzymatically treated and non-treated, along with fungal mycelium; 5 g of wet weight) was removed aseptically from the flasks after incubation for 10 d and then suspended in 20 mL of acetate buffer (20 mmol/L, pH 5.0) and agitated at 200 rpm for 1 h. The extrudates were pressed through a muslin cloth to maximize enzyme extraction and centrifuged at 10,000 rpm for 10 min at 4°C. The obtained enzyme solution was assayed to determine the activities of different enzymes.

Laccase (E.C. 1.10.3.2) activity was determined using the method described by Paavola et al. [18]. MnP (E.C. 1.11.1.13) activity was measured by oxidation of DMP (2, 6-dimethoxy phenol) in sodium tartrate buffer 0.1 M (pH 4.5) in the presence of H_2_O_2_ and MnSO_4_ to coerulignone. The increase in absorbance at 469 nm was determined at 30°C and the activity was calculated using an ϵ469 of 27,500 /mol/cm for DMP [19]. Xylanase activity was determined by measuring the release of reducing sugars from birchwood xylan (1.0% w/v) using the dinitrosalicylic acid method [20].

### Proximate and chemical composition of WS

Dried WS samples were analyzed in triplicate for moisture, dry matter, ether extracts, crude fiber, total carbohydrates, CP, and total ash content [21]. The acid detergent fiber (ADF), neutral detergent fiber (NDF), cellulose, hemicellulose, and lignin contents were determined using the methods described by Van Soest et al. [22,23]. The C/N ratios in the fermented samples were estimated using a CHNS analyzer (Elementar Vario EL III; Analysensysteme GmbH, Hanau, Germany). Percent efficiency of the solid state fermentation (SSF) process was expressed as the loss of lignin content compared to carbohydrate breakdown and calculated using the method described by Moyson and Verachtert [24]. Loss of WS components after treatment was calculated using the following formula:

$$U1=[\left(G_{0}C_{0-}G_{1}C_{1}/G_{0}C_{0}\right]\times 100$$

Where, G0 and G1 are the masses of dry substrates at the beginning and end of fermentation, respectively, and C0 and C1 are the dry weights of the components at the beginning and end of fermentation, respectively.

### *In vitro* digestion

*In vitro* digestion of non-inoculated and fungal-treated WS was estimated according to the methods described by Akhter et al. [25] with some modifications. Briefly, the inoculum was prepared by mixing fresh buffalo fecal matter (100 g/L) in pre-warmed (39°C) artificial saliva containing NaHCO_3_ (9.80 g), Na_2_HPO_4_•7H_2_O (7.0 g); KCl (0.57 g), NaCl (0.47 g), MgSO_4_•7H_2_O (0.12 g), and 1.0 mL of CaCl_2_ (4% w/v). The solution was filtered through a muslin cloth and a 500-mg aliquot of the wheat straw was placed in a 50-mL centrifuge tube and the prepared fecal suspension was added. Then, CO_2_ gas was flushed into the centrifuge tubes for 48 h in a 39°C water bath. The mixture contained in the centrifuge tubes was centrifuged and the supernatant was discarded. Next, 35 mL of acidified pepsin (6.6 g in 1 L of 0.1 mol/L HCl) was added to the pellet and the tubes were again incubated under similar conditions for 48 h and then centrifuged. The obtained pellet was filtered through tared filter paper and dried. *In vitro* digestion was expressed as the loss of dry matter mass during incubation under standard assay conditions.

## Results

A previous study reported that the selected soil isolate, identified as *Penicillium charlesii*, produced 61.4 IU/mL in 54 h in 220 L of statistically optimized medium composed of (% w/v) TA (2.0), sucrose (1.0), NaNO_3_ (1.0), and MgSO_4_ (0.08; pH 5.0) contained in 300-L vessel and then fed with 1.0% (w/v) TA after 24 h of incubation. The tannase obtained from the fermentation broth was partially purified by ultrafiltration (30 kDa), ammonium sulfate precipitation, and then lyophilized with a final yield of 5.8 IU/mg [26].

**Figure 1:**
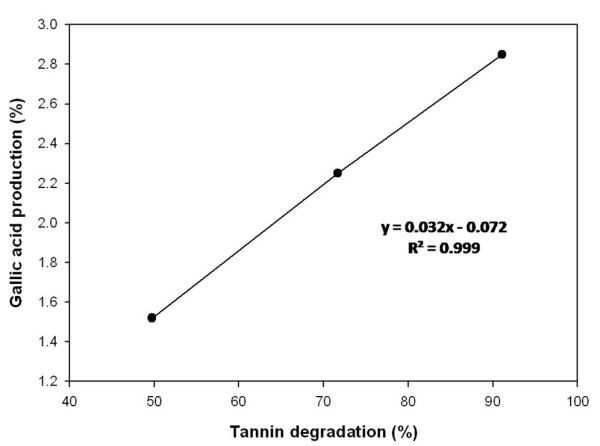
**Positive correlation between gallic acid production and tannic acid degradation (R**^**2**^ **= 0.999).**

Analysis of the raw WS (control) and tannase-treated WS samples (at 0.1% w/w of tannase concentration) revealed tannin contents of 3.6% and 1.8% (w/w), respectively. On further increase in tannase concentration up to 0.3% (w/w), a 1.8-fold higher reduction in tannin content was noted compared to the results obtained at 0.1% tannase. These results clearly indicated that an increased tannase concentration resulted in a decrease in tannin content (Table 1). There was a very strong positive correlation (R^2^ = 0.999) between enhanced GA production and TA degradation (Figure 1).

**Table 1:** Enzymatic tannin removal from wheat straw at different concentration of tannase

**Tannase concentration**	**Tannase activity, IU**	**Remaining tannin, g/100 g WS**	**Tannin degradation, %**	**Gallic acid produced, g/100 g WS**
Raw wheat straw (control)	0.0 ± 0.00	3.60 ± 0.05 (100%)	0.0 ± 0.00	0.0 ± 0.00
WS + tannase (0.1%)	423 ± 11.6	1.80 ± 0.04 (50.3%)	49.7 ± 0.99	1.5 ± 0.03
WS + tannase (0.2%)	846 ± 27.5	1.02 ± 0.01 (28.3%)	71.7 ± 1.79	2.2 ± 0.06
WS + tannase (0.3%)	1269 ± 47.6	0.33 ± 0.002 (8.9%)	91.1 ± 2.7	2.8 ± 0.09

WS = wheat straw.

**Figure 2:**
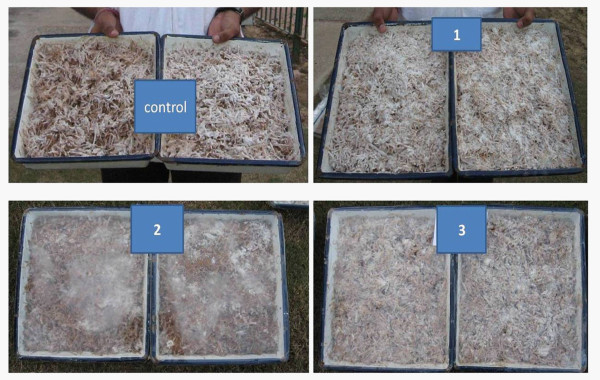
**Growth pattern of *Ganoderma *** **sp. on raw wheat straw and tannin free ssf treated wheat straw (TFWS) Control: raw wheat straw; 1: TFWS treated with 0.1% tannase; 2: TFWS treated with 0.2% tannase; 3: TFWS treated with 0.3% tannase.**

After tannin removal, tannin-free WS was then subjected to SSF using *Ganoderma* spp. to further improve the WS composition and to analyze the various enzymes produced and substrates transformed during fungal fermentation (Figure 2).

As in the previous experiment (Table 1), an increase in tannase concentration resulted in incremental tannin degradation. But *Ganoderma* spp. grown on tannin-free WS showed that 0.1% (w/w) tannase was the optimal concentration for a remarkable increase in fungus-produced lignocellulolytic enzymes. Our results also indicated that at the optimal concentration of 0.1% tannase (w/w) resulted in a 1.12-fold increase in laccase activity, a 1.06-fold increase in MnP activity, and a 1.17-fold increase in xylanase activity after incubation for 10 d compared to raw untreated WS (Table 2). Along with the production of various lignocellulolytic enzymes, the *Ganoderma* spp. could also colonize and rapidly degrade WS, thereby showing a variation in dry matter loss (DML). A higher DML of 14.6% (w/w) was obtained in *Ganoderma* grown on WS treated with 0.1% (w/w) tannase. By further increasing the tannase concentration beyond 0.1%, a decline in the lignocellulolytic activities of MnP, xylanase, and laccase along with lower DML was observed. Based on these results, we hypothesized that due to the relatively poor growth of *Ganoderma*, as observed at higher tannin degradation or GA concentrations; lignocellulolytic activity was decreased, indicating a reduction in lignin depolymerization.

**Table 2:** Enzyme production and substrate transformation during fungal fermentation of wheat straw

**Tannase concentration**	**Treated with *Ganoderma *** **sp.**		
	**Laccase, U/g**	**MnP, U/g**	**Xylanase, U/g**
Raw wheat straw	4012 ± 160.4	126 ± 2.8	221 ± 6.0
WS + tannase (0.1%)	4518 ± 203.3	133.2 ± 3.6	260 ± 7.8
WS + tannase (0.2%)	3216 ± 120.6	116 ± 2.0	210 ± 5.2
WS + tannase (0.3%)	2213 ± 60.8	56.21 ± 0.84	139 ± 2.4

WS = wheat straw.

The results of WS compositional analysis presented in Table 3 clearly showed a higher percentage of degraded lignin (37.8% w/w) with a lower L/C ratio of 0.18 was obtained in the tannin-free *Ganoderma*-treated WS with 0.1% (w/w) tannase. In addition, the percentage of degraded hemicellulose decreased to 17.9% (w/w), whereas a further increase in tannase concentration (up to 0.3%, w/w) resulted in a lower concentration of degraded lignin (13.0%, w/w) with increased cellulose degradation (up to 24.5%, w/w) and a higher L/C ratio of 0.27. Also, 32.2% of the *in vitro* digestibility using 0.1% tannase-treated myco-straw; therefore, 0.1% (w/w) tannase-treated myco-straw was selected, as it showed a lower L/C ratio along with a decent percentage of *in vitro* digestion, which indicated improved quality of cattle feed.

**Table 3:** **Disappearance (%) of cell wall components in raw wheat straw and enzyme treated wheat straw subjected to treatment with *Ganoderma *** **sp.**

**Substrate**	***In vitro *** **digestibility,%**	**% Degradation**							L/C ratio*
		**Wt**	**ADF**	**Lignin**	**Cellulose**	**NDF**	**HC**	**CP**	
SSF treated WS (myco- straw)	26.8 ± 0.8	11.6 ± 0.2	17.3 ± 0.4	24.0 ± 0.5	22.6 ± 0.6	18.9 ± 0.4	21.7 ± 0.5	2.1 ± 0.02	0.23 ± 0.003
Tannin free ssf treated WS (0.1%)	32.2 ± 1.1	14.6 ± 0.3	20.3 ± 0.5	37.8 ± 1.3	20.4 ± 0.4	19.5 ± 0.4	17.9 ± 0.3	2.7 ± 0.04	0.18 ± 0.001
Tannin free ssf treated WS (0.2%)	27.9 ± 0.7	12.4 ± 0.2	14.2 ± 0.3	16.6 ± 0.3	21.9 ± 0.5	19.3 ± 0.2	29.6 ± 0.8	2.14 ± 0.03	0.25 ± 0.004
Tannin free ssf treated WS (0.3%)	24.2 ± 0.4	10.9 ± 0.1	10.1 ± 0.1	13.0 ± 0.2	24.5 ± 0.7	19.6 ± 0.3	39.1 ± 1.4	2.13 ± 0.03	0.27 ± 0.005

ADF Acid detergent fiber, NDF Neutral detergent fiber, HC Hemicellulose, CP Crude protein; * data calculated on as such basis (w/w); Uninoculated wheat straw contains *in vitro* digestibility of 146 g/kg (14.6%).

*Ganoderma*-treated WS (myco-straw) exhibited maximum SSF efficiency (approximately 20%) on incubation day 10. A higher SSF efficiency leads to better nutrient assimilation by fungi and a consistent increase in CP content. It was observed that a higher efficiency of 24.4% was obtained in tannin-free *Ganoderma*-treated WS (0.1% tannase, w/w) on the 10th day of incubation compared to the untreated control WS and 1.22-fold higher compared to myco-straw. Furthermore, the tannin-free *Ganoderma*-treated WS at a tannase concentration of 0.1% (w/w) showed a 1.28-fold higher CP content compared to myco-straw and a 25.0% lower C/N ratio in the fermented substrate (Table 4).

**Table 4:** CP of wheat straw

**Substrate**	**N**	**C**	**S**	**H**	**CP**	**C/N ratio**
SSF treated WS	0.33 ± 0.007	36.5 ± 1.00	0.24 ± 0.003	3.83 ± 0.07	2.09 ± 0.02	109.16 ± 3.54
Tannin free ssf treated WS (0.1%)	0.43 ± 0.01	34.9 ± 0.9	0.48 ± 0.007	4.89 ± 0.11	2.66 ± 0.4	81.88 ± 2.04
Tannin free ssf treated WS (0.2%)	0.342 ± 0.008	28.7 ± 0.5	0.24 ± 0.004	3.22 ± 0.05	2.14 ± 0.03	83.80 ± 2.30
Tannin free ssf treated WS (0.3%)	0.341 ± 0.008	29.3 ± 0.6	0.29 ± 0.005	4.09 ± 0.09	2.13 ± 0.03	86.04 ± 2.58

Crude Protein (CP) content in raw wheat straw: 0.56; N: nitrogen, C: carbon; S: sulphur; H: hydrogen.

## Discussion

Worldwide energy and environmental crises have forced the re-evaluation of efficient utilization of existing resources or to find alternative natural and renewable resources in nature, particularly to dispose of organic waste, using clean technologies. Every year, large quantities of cellulosic agriculture byproducts are being produced. In the face of global industrialization and the subsequent environmental consequences, as exemplified by the decline in forest belts and global climate changes, forces us to consider the potential benefits of finding alternative sources of cellulose. To date, WS is rarely used for cattle feed because of its relatively low nutritional value, particularly the low protein content, high fiber content, and low digestibility by ruminants. Thus, improvements in the nutrient availability in cattle feed presents a significant challenge to animal nutritionists.

The use of fungi or fungal enzymes that can metabolize lignocellulose presents a potential biological method to improve the nutritional value of WS by selective delignification [11]. The use of tannase or microbes that produce this enzyme in feed preparations increases the bioavailability of nutrients by hydrolyzing phenols, which acts as anti-nutritional factors [27]. However, due to high production costs and our limited knowledge of the catalytic activities of tannase, this enzyme is currently used under very few circumstances.

The concentration of tannins in feed plays a vital role on the digestibility by ruminants [28]. The highest tannin content showed the lowest digestibility, thus showing a strong negative correlation (r = -0.993) [12].

There are several reports in the literature confirming that WS contains appreciable amounts of tannins [29,30]. Enhancement in the *in vitro* digestibility of WS up to 2.2-fold was observed by treatment with 0.1% tannase (w/w), as well as WRF (*Ganoderma* sp.) under SSF, as compared to the digestibility of untreated WS. Similar to our results, Arora and Sharma [12] reported enhanced *in vitro* digestibility up to 1.66-fold by WS treatment with the fungus *Phlebia brevispora*, obtained from northwestern and northeastern regions of India, while a 1.78-fold increase was observed by the use of fungi obtained in central India. In the present investigation, we established that the presence of 0.1% (w/w) tannase enhanced lignin degradation as well as *in vitro* digestibility.

Regarding fibrolytic enzymes, several researchers have reported that the presence of aromatic compounds was required, to a certain extent, to enhance ligninase production in several WRF for lignin degradation. In the present study, it was noted that at higher tannase concentrations beyond optimal, more toxic phenolic compounds with anti-nutritional properties (tannin) were degraded up to approximately 90% with 0.3% (w/w) tannase along with the synthesis of higher amounts of GA, which is comparably less toxic than NA. However, this increased GA concentration could lead to lower activity of different lignocellulolytic enzymes produced from *Ganoderma* spp. Therefore, an optimal GA concentration is required, as GA acts as an inducer, not an inhibitor, of the lignocellulytic enzymes produced from WRF. In the present report, the amount of GA produced (1.5 g/100 g) by tannin degradation with 0.1% (w/w) tannase was sufficient to promote growth of *Ganoderma* spp. and to induce production of lignocellulytic enzymes. Above this concentration, GA acts as an inhibitor of the lignocellulytic enzymes laccase, and MnP produced by WRF, which are primarily responsible for lignin degradation through inhibition of *Ganoderma* growth. Our results are in accordance with those reported by Gnanamani et al. [31], in which the presence of GA at an optimal concentration of 300 mmol/L could induce activity of the lignocellulytic enzyme laccase in *Phanerochaete chrysosporium* (NCIM 1197). Also, Patel et al. [32] reported an increase in laccase activity of up to 1.3-fold in the presence of an optimal GA concentration. Contrary to our results, Cavallazzi et al. [33] observed no laccase activity in the presence of catechol and GA, even after 18 d of cultivation. The optimal GA concentration for luxurious growth and enhanced enzyme levels in WRF might produce toxic effects to ruminants by feed digestion, although the effect of toxicity will definitely be less than that induced by tannins. Lin et al. [34] postulated that produced GA can easily be assimilated by ruminants due to the sequential actions of different enzymes present in the rumen microflora to pyrogallol, phloroglucinol, and to the less toxic compounds acetate and butyrate.

In the present study, we found that by increasing the tannase concentration beyond optimal (0.1% w/w), a greater amount of tannin was degraded and converted into GA and glucose in WS. Therefore, tannin-free *Ganoderma*-treated WS (beyond 0.1% w/w tannase) contains a lower tannin concentration, which is generally associated with or forms complexes with the hemicellulases of WRF, thereby leading to more free sites available for hemicellulases to degrade greater amounts of hemicellulose in WS by the actions of WRF. On compositional analysis of WS, it was observed that greater amounts of hemicellulose were degraded as compared to cellulose at higher tannase concentrations (beyond 0.1% w/w tannase). Similar results have been reported by several researchers who found that tannin-rich agro-material can easily inhibit hemicellulases over cellulases through association, as cellulases are generally associated with the cell walls of WRF, while hemicellulases are extracellular and are, therefore, more sensitive to tannins present in lignocellulosic materials. In this respect, our results are in accordance with those reported by Hervás et al. [35], who observed increased degradation of cellulose as compared to hemicelluloses in the presence of higher residual tannins (i.e., at lower tannase concentrations).

The lignin to cellulose ratio (L/C ratio) is considered important for cattle feed. This ratio clearly indicates that during treatment greater quantities of lignin are degraded, thereby leaving more cellulose in the residual substrate. Therefore, enzyme-treated feed may contain more cellulose as compared to lignin in residual substrates that can be easily digested and also provides a rich source of energy to ruminants. The L/C ratio of the tannin-free *Ganoderma*-treated WS was 0.18 at a tannase concentration of 0.1% (w/w). Calzada et al. [36] observed lignin degradation in the range of 24% to 55% (w/w), whereas cellulose degradation ranged from 34% to 42% (w/w) for WS inoculated with the Pleurotus spp. of WRF. Basu et al. [6] reported that the maximum desirability coefficient after incubation for 6 d was 19.5% (w/w) lignin and 17.8% (w/w) cellulose degradation, although the fungus continued to degrade the substrate thereafter. Fungal fermentation using *Ganoderma* sp. rckk02 decreased the lignin content of WS by 34.95% (w/w) with a subsequent reduction in cellulose content by 34.33% (w/w) after incubation for 15 d [37].

A 78.6% (w/w) increase in CP content was observed in tannin-free *Ganoderma*-treated WS with 0.1% (w/w) tannase as compared to raw WS. While, a 27.3% (w/w) higher CP content was obtained in *Ganoderma*-treated WS (myco-straw). Various studies on fungal fermentation have reported the enrichment of WS with fungal biomass as the protein source under SSF [14,38]. While, Srivastava et al. [13] reported a 57.11% (w/w) increase in CP content by fermenting WS with *Ganoderma* sp. rckk02 compared to raw WS (control). The increase in CP content is partially attributable to fungal proteins, particularly chitin [38], although the observed decrease in certain cases cannot be fully explained. The C/N ratio is an important marker of biomass pretreatment because degradation of lignocellulosic material is dependent on the C/N ratio of the material. Deshpande et al. [39] reported that an optimal C/N ratio promotes good fungal growth and, consequently, higher enzyme production.

It could be concluded that the use of a combination of various lignocellulytic enzymes present in WRF and tannase obtained from *Penicillium charlesii* resulted in complete fermentation over a short period, which indicates the scope of enriched feed production at an industrial scale. The biological treatment processes are safe, eco-friendly, easy to perform, and involves lower operational costs as compared to other treatment methods. Besides, in this process, the inoculum could be raised from existing growing cultures and reused several times to inoculate bulk amounts of residues. While, the use of chemicals is limited because once used, these substances leach into water sources, thereby requiring further treatments involving large amounts of other chemicals. If this technology could be commercialized in the future, then the enzyme tannase (lyophilized or liquid form) along with the *Ganoderma* sp. (dried or lyophilized form) can be made available to cattle ranchers as enriched feed at costs subsidized by the government.

## Competing interests

All authors declare that they have no competing interests.

## Authors’ contribution

SR and SM designed and performed the experiments, including chemical analysis, collected and analyzed the data, and wrote the manuscript. RKS verified the validity of the experimentation and checked the results. All of the authors read and approved the final version of this manuscript.
